# Simultaneous Multioutcome Synthesis and Mapping of Treatment Effects to a Common Scale

**DOI:** 10.1016/j.jval.2013.12.006

**Published:** 2014-03

**Authors:** Guobing Lu, Daphne Kounali, A.E. Ades

**Affiliations:** School of Social & Community Medicine, University of Bristol, Bristol, UK

**Keywords:** congeneric tests, cross-walking, mapping, multioutcome synthesis

## Abstract

**Objectives:**

A new method is presented for both synthesizing treatment effects on multiple outcomes subject to measurement error and estimating coherent mapping coefficients between all outcomes. It can be applied to sets of trials reporting different combinations of patient- or clinician-reported outcomes, including both disease-specific measures and generic health-related quality-of-life measures. It is underpinned by a structural equation model that includes measurement error and latent common treatment effect factor. Treatment effects can be expressed on any of the test instruments that have been used.

**Methods:**

This is illustrated in a synthesis of eight placebo-controlled trials of TNF-α inhibitors in ankylosing spondylitis, each reporting treatment effects on between two and five of a total six test instruments.

**Results:**

The method has advantages over other methods for synthesis of multiple outcome data, including standardization and multivariate normal synthesis. Unlike standardization, it allows synthesis of treatment effect information from test instruments sensitive to different underlying constructs. It represents a special case of previously proposed multivariate normal models for evidence synthesis, but unlike the former, it also estimates mappings. Combining synthesis and mapping as a single operation makes more efficient use of available data than do current mapping methods and generates treatment effects that are consistent with the mappings. A limitation, however, is that it can only generate mappings to and from those instruments on which some trial data exist.

**Conclusions:**

The method should be assessed in a wide range of data sets on different clinical conditions, before it can be used routinely in health technology assessment.

## Introduction

The effects of new treatments in randomized controlled trials are often measured by test instruments that record patient- or clinician-reported “subjective” outcomes. Typically, there are a range of test instruments available to investigators. For example, the efficacy of treatments for depression may be evaluated by the Hamilton [1], Beck [2], or Montgomery-Asberg scales [3]. These would all be regarded as measuring *approximately* the same underlying construct. In dermatological or rheumatic illnesses, or for many cancers, there is also a wide range of patient- or clinician-reported instruments available, but most are designed to measure different disease-related constructs. In ankylosing spondylitis, for example, randomized trials routinely investigate treatment effects on pain, using a numeric rating scale or a continuous visual analogue scale (VAS); on disease progression, using the Bath Ankylosing Spondylitis Disease Activity Index [4]; and on patients’ daily life, using the Bath Ankylosing Spondylitis Functional Index [5].

One can further distinguish between the above disease-specific measures (DSMs) and generic health-related quality-of-life (HRQOL) instruments that are designed to be applied to almost any condition, such as the Euroqol five-dimensional (EQ-5D) questionnaire [6] and the multipurpose short-form 36 health survey [7].

The existence of so many test instruments raises a number of issues in meta-analysis, the statistical pooling of treatment effects reported in different trials on the same treatments [8], [9], [10]. Several different approaches have been described. S*tandardization* (division of treatment effects by the sample SD) allows synthesis of different instruments on a common scale [11]. A disadvantage is that division by the sample standard error can only add to heterogeneity. It also assumes that all the measures are equally sensitive to the treatment effect. *Composite outcomes* can be created through linear combinations of treatment effects on different instruments [9], [10], [11], [12], although these are seldom used because investigators prefer outcomes to be measured on familiar scales. Various forms of *multivariate meta-analysis* based on within- and between-trial correlation [13], [14], [15], [16], [17], [18] have also been proposed. These approaches have different properties, objectives, and scope of application: we return to discuss them in greater detail later.

A second, quite different, problem is the “mapping” from treatment effects on DSMs to treatment effects on generic HRQOLs. This is widely used in health technology assessment (HTA), when estimates of treatment effects on generic HRQOL instruments are required in cost-effectiveness analyses, but treatment effect data are available only on DSMs. Usually, an externally sourced mapping coefficient is used to translate the treatment effect on a DSM into a treatment effect on a generic HRQOL scale such as the EQ-5D questionnaire [19], [20]. These mappings are usually derived from a regression based on an external “estimation” dataset. The regression equation is then applied to “source” (DSM) estimates to generate “target” (generic HRQOL) estimates, at the level of either a mean effect or individual patient data [20], [21]. We will return to consider the way mappings are derived and used in HTA in the discussion.

This article presents a method for multioutcome synthesis based on the hypothesis that for a defined population of patients undergoing a given type of treatment, mapping coefficients, defined as the *ratios* of the true treatment effects$\delta_{jr},\;\delta_{js},\;\delta_{jt}$ on instruments *r*, *s*, *t*, remain approximately constant across trials *j*:

(1) $$\frac{\delta_{js}}{\delta_{jr}}=\beta_{r->s}$$

It follows from this definition that mappings are invertible and transitive [22].

(2) $$\beta_{r->s}=\frac{1}{\beta_{s->r}},\;\mathrm{and}\;\beta_{r->s}\beta_{s->t}=\beta_{s->t}$$

The advantage of the proposed method over other forms of multioutcome synthesis, and over previous methods for mapping, is that simultaneously it “borrows strength” across correlated outcomes, both within and between trials, it allows investigators to express the pooled estimates of treatment effects on any scale, but without introducing further heterogeneity, and it estimates the mapping coefficients between treatment effects, subject to invert ability and transitivity constraints, as well as estimates treatment effects that are consistent with the mappings.

We begin by describing an illustrative data set, and then describe the statistical methods, followed by a short description of the results. In discussion, we contrast the proposed model with existing approaches to multioutcome synthesis and with current approaches to mapping in HTA. Some technical details can be found in appendices, available as Supplemental Material found at doi:10.1016/j.jval.2013.12.006.

## Methods

### Materials: TNF-α Inhibitors in Ankylosing Spondylitis

The manufacturers of the TNF-α inhibitor golimumab (Simponi) submitted a cost-effectiveness analysis of the product in the treatment for ankylosing spondylitis to the National Institute for Health and Clinical Excellence in November 2010 [23]. The submission included network meta-analyses [24] of several placebo-controlled trials of the TNF-α inhibitors golimumab, etanercept, infliximab, and adalimumab. Eight trials [25], [26], [27], [28], [29], [30], [31], [32] reported between one and five of the following six test instruments: Pain on a VAS; Bath Ankylosing Spondylitis Functional Index, Bath Ankylosing Spondylitis Disease Activity Index, the Ankylosing Spondylitis Quality of Life scale [33], [34], and the short-form 36 health survey physical and mental components summaries. We extracted the mean change scores on each arm (follow-up assessment minus baseline score) and the standard error of the change scores on their original scales (see Appendix A in Supplemental Materials found at doi:10.1016/j.jval.2013.12.006). If results were reported at more than one follow-up time, we chose just the first. The final data set is shown in Table 1. One study was a three-arm trial.

**Table 1:** Treatment effects on change from baseline, relative to placebo, and their standard errors in eight trials of TNF-α inhibitors in ankylosing spondylitis.*

**Trial**	Treatment	**Weeks**	**PAIN-VAS**	**BASFI**	**BASDAI**	**SF-36 PCS**	**SF-36 MCS**	**ASQOL**
1. Gorman (2002) [25]	ETA	16	−4.15 (0.803)	−2.2 (0.772)				
2. Brandt (2003) [26]	ETA	6		−1.7 (0.80)	−2.2 (0.553)			
3. Davis (2003) [27]	ETA	24	−1.83 (0.410)	−1.41 (0.285)	−1.91 (0.258)			
4. van der Heijde (2006) [28]	ADA	12	−1.956 (0.350)	−1.414 (.267)	−1.8 (0.283)			
5. Braun (2002) [29]	INF	12		−2.0 (0.561)	−2.6 (0.433)	8.3 (5.74)	3.85 (4.33)	
6. van der Heijde (2005) [30]	ADA	24		−1.5 (0.236)	−1.8 (0.286)	5.2 (1.026)	1.6 (1.162)	−2.4 (0.488)
7. van der Heijde (2009) [31]	INF	24	−2.6 (0.333)	−1.7 (0.230)	−2.5 (0.274)	9.4 (0.957)	0.7 (1.045)	
8. Inman (2008) [32]	GOL 50	12	−2.7 (0.412)	−1.5 (0.264)		4.9 (1.164)	1.4 (1.023)	
8. Inman (2008) [32]	GOL 100	12	−2.8 (0.424)	−1.6 (0.274)		6.0 (1.069)	3.6 (1.252)	

ADA, adalimumab 40 mg; ASQOL, Ankylosing Spondylitis Quality of Life scale; ETA, etanercept 25 mg; BASDAI, Bath Ankylosing Spondylitis Disease Activity Index; BASFI, Bath Ankylosing Spondylitis Functional Index; GOL 50, golimumab 50 mg; GOL 100, golimumab 100 mg; INF, infliximab 5 mg; SF-36 MCS, short-form 36 health survey mental component summary; SF-36 PCS, short-form 36 health survey physical component summary; VAS, visual analogue scale.

⁎ ASQOL, 0 to 18 scale; BASDAI, 0 to 10 scale; BASFI, 0 to 10 scale; PAIN-VAS, 0 to 10 scale; SF-36 PCS and SF-36 MCS, 0 to 100 scale.

Our methods require information on the between-test within-study correlations. For this purpose, we used the Evaluation of Ankylosing Spondylitis Quality of Life cohort study [33], [34]. This examined 612 individuals suffering from ankylosing spondylitis, using the six instruments reported in the randomized controlled trials.

### Common Treatment Factor Model

A common factor model [22], [35] provides the underlying rationale for our approach. Consider data on individuals *t* randomized to an active treatment in trial *j* and individuals *c* randomized to placebo. Two outcomes are observed, measured by instruments *r* and *s*. We can express the observed patient outcomes *Y*_*r*_ and *Y*_*s*_ on these instruments in terms of a standardized common latent variable *y* and error terms $\varepsilon_{r},\;\varepsilon_{s}\;⊥\;y$that are orthogonal to *y* but not necessarily to each other:

(3) $$Y_{jcr}=a_{r}+b_{r}y_{c}+c_{r}\varepsilon_{jcr}Y_{jtr}=a_{r}+b_{r}(y_{t}+\delta_{j})+c_{r}\varepsilon_{jtr}Y_{jcs}=a_{s}+b_{s}y_{c}+c_{s}\varepsilon_{jcs}Y_{jts}=a_{s}+b_{s}(y_{t}+\delta_{j})+c_{s}\varepsilon_{jts}Y_{c},\;Y_{t},\;\varepsilon_{jcr},\;\varepsilon_{jtr},\;\varepsilon_{jcs},\;\varepsilon_{jts}\sim N(0,\;1)$$

The coefficients ($a_{r},\;a_{s}$) are the arbitrary intercepts, and $b_{r},\;c_{r},\;b_{s},\;c_{s}$ are factor loadings for the latent variable *y* and error terms $\varepsilon_{r},\;\varepsilon_{s}$ on each scale. The factor *y* represents the common *treatment construct*, the component of the test that is sensitive to treatment. The specific error components $\varepsilon_{r},\;\varepsilon_{s}$comprise both measurement error and other components that are (by definition) insensitive to treatment. A treatment effect $\delta_{j}$ on the common latent factor *y* will manifest as a treatment effect $b_{r}\delta_{j}$on instrument *r* and $b_{s}\delta_{j}$on instrument *s*. The mapping coefficient from *r* to *s* is therefore $\beta_{r->s}=b_{s}/b_{r}$. Clearly, mappings derived from this model have the properties in (2). If the error variables $\varepsilon_{r},\;\varepsilon_{s}$ were also orthogonal, then *r* and *s* would qualify as *congeneric* tests [36] in a classical measurement theory [37] formulation. Note the implication that the mapping ratio will remain constant as $\delta_{j}$varies from trial to trial. This assumption can be relaxed. Model (3) could be extended to a case in which there are separate treatment effects on *k* orthogonal, treatment-sensitive constructs, *y*_1_, *y*_2_, _…_, *y*_*k*._ Although the mapping coefficients will now be ratios of linear functions of loadings, they will still be estimated by ratios of treatment effects.

The common factor model (3) plays no direct role in the meta-analytic approach to mapping and synthesis we now describe, but it provides an underlying rationale for our approach to mapping, and explains why it leads to a synthesis with different properties to other multivariate methods [13], [14].

### Data Likelihood

In each trial *j*, for an instrument *r*, the mean outcomes at follow-up and at baseline are, dropping the subscript *j,* ${\bar{Y}}_{r\mathrm{TF}}$ and ${\bar{Y}}_{r\mathrm{TB}}$, respectively, in the treatment arm and ${\bar{Y}}_{r\mathrm{CF}}$, and ${\bar{Y}}_{r\mathrm{CB}}$, in the control arm, with variances $V_{r\mathrm{TF}},\;V_{r\mathrm{TB}},\;V_{r\mathrm{CF}},\;V_{rCB}$ and sample sizes *n*_*T*_ and *n*_*C*_. What is reported varies somewhat from trial to trial. The variances and covariances [38] of the mean treatment effects on change scores on instruments *r* and *s* are as follows:

(4) $${\hat{D}}_{m}=({\bar{Y}}_{m\mathrm{TF}}-{\bar{Y}}_{m\mathrm{TB}})-({\bar{Y}}_{m\mathrm{CF}}-{\bar{Y}}_{m\mathrm{CB}}),\;S_{mm}=Var({\hat{D}}_{m})=\frac{V_{mT}}{n_{T}}+\frac{V_{mC}}{n_{C}},S_{rs}=Cov({\hat{D}}_{r},\;{\hat{D}}_{s})=\rho_{rs}\left(\frac{\sqrt{V_{rT}V_{sT}}}{n_{T}}+\frac{\sqrt{V_{rC}V_{sC}}}{n_{C}}\right)$$

where *ρ*_*rs*_ is the correlation between *change scores* on instruments *r* and *s.* In trials in which the variance of the change scores on each arm, *V*_*mT*_, *V*_*mC*_, is not reported, we have used the variances at baseline and follow-up, following the common practice of assuming a 0.5 correlation between baseline and follow-up scores, on every outcome [39].

None of the trials report correlations between test instruments. We show (Appendix B in Supplemental Materials found at doi:10.1016/j.jval.2013.12.006) that if we assume Corr(*Y*_*rB*_*,Y*_*sF*_|*Y*_*sB*_) = 0, meaning that the correlation between *Y*_*rB*_ and *Y*_*sF*_ comes *only* through the correlation between *Y*_*rB*_ and *Y*_*sB*_, then the correlation between change scores is equal to the correlation between cross-sectional scores. Information on the latter was made available from the Evaluation of Ankylosing Spondylitis Quality of Life study [33] (Table 2). Sensitivity analyses were run to assess the effect of increasing or decreasing *ρ*_*rs*_ by 10%.

**Table 2:** Correlation matrix from the EASi-QoL study, based on 571 patients with ankylosing spondylitis with complete data.

Outcomes	**Pain-VAS**	**BASFI**	**BASDAI**	**ASQOL**	**SF-36 PCS**	**SF-36 MCS**
Pain-VAS	1	0.703	0.852	0.738	−0.668	−0.493
BASFI	0.703	1	0.811	0.829	−0.842	−0.463
BASDAI	0.852	0.811	1	0.856	−0.751	−0.583
ASQOL	0.738	0.829	0.856	1	−0.785	−0.654
SF-36 PCS	−0.668	−0.842	−0.751	−0.785	1	0.339
SF-36 MCS	−0.493	−0.463	−0.583	−0.654	0.339	1

ASQOL, Ankylosing Spondylitis Quality of Life scale; BASDAI, Bath Ankylosing Spondylitis Disease Activity Index; BASFI, Bath Ankylosing Spondylitis Functional Index; EASi-QoL, Evaluation of Ankylosing Spondylitis Quality of Life; SF-36 MCS, short-form 36 health survey mental component summary; SF-36 PCS, short-form 36 health survey physical component summary; VAS, visual analogue scale.

The likelihood of a treatment effect on a single outcome on instrument *m* in the two-arm trial *j* can therefore be represented as ${\hat{D}}_{jm}\sim N(\delta_{jm},\;S_{j,mm})$. If we consider the case in which *M* outcomes are reported, 2≤M≤6, this has a multivariate normal likelihood, ${\hat{D}}_{j}\sim MVN(\delta_{j},\;S_{j})$. The diagonal elements for instrument *r* are $S_{j,rr}$, and the off-diagonal elements for instruments *r* and *s* are $S_{j,rs}$, as defined in (4). The likelihood for multiarm trials is shown in Appendix C in Supplemental Materials found at doi:10.1016/j.jval.2013.12.006.

### Models for Treatment Effects and Mappings

Pain-VAS was chosen (arbitrarily) as the “baseline” test instrument, indexed *m* = 1. The model for the treatment effect on instrument 1 in trial *j* is a standard random-effects model, $\delta_{j1}\sim N(\mu_{1},\;\sigma_{1}^{2})$. In a three-arm trial comparing treatment *h* and *k* with placebo, the treatment effects relative to placebo, $\delta_{j,k,1}$ and $\delta_{j,h,1}$, are correlated. Assuming homogeneous variances [40], [41], the treatment effects relative to placebo have a multivariate normal distribution:

(5) $$\left(\begin{array}{l} \delta_{j,h,1}\\ \delta_{j,k,1} \end{array}\right)\sim ΜVΝ\left(\left(\begin{array}{l} \mu_{1}\\ \mu_{1} \end{array}\right)\left(\begin{array}{ll} \sigma_{1}^{2} & \sigma_{1}^{2}/2\\ \sigma_{1}^{2}/2 & \sigma_{1}^{2} \end{array}\right)\right)$$

In a Bayesian framework, we assign vague priors to the hyperparameters: *μ*_1_ ~ *N*(0, 100^2^) and *σ*_1_ ~ *U*(0, 10).

To map treatment effects on each of the *M* instruments into treatment effects on every other instrument, *M*(*M* − 1)/2 mappings must be estimated. Because of the constraints embodied in Equation 2, (*M* − 2)(*M − 1*)*/*2 of these can be defined from the remaining *M* − 1. This makes it possible to identify all 15 mappings from the eight trials. We specify the mappings from Pain-VAS to the five other instruments as “basic” [42] parameters that are assigned vague priors, while the remaining 10 mappings are “functional” parameters defined in terms of the five basic ones. Note that the relative signs of the mappings, reflected in the correlations (Table 2), are considered “known.”

(6) $$\beta_{1\to 1}=1\beta_{1\to m}^{}\sim N(0,100^{2}),\;\beta_{1\to m}=\mathrm{Sign}(\mathrm{Abs}(\beta_{1\to m}^{})),\;m=2\ldots M\beta_{r\to s}=\beta_{r\to 1}\beta_{1\to s}=\frac{\beta_{1\to s}}{\beta_{1\to r}},\;r=2\ldots (M-1),\;s=(r+1)\ldots M$$

This model (6) is a “fixed mapping” model. In the event, this did not fit the data well, a random mapping model was constructed, in which the mapping coefficient $\beta_{j,r\to s}$ applying in any trial *j* is drawn from a normal distribution:

(7) $$\beta_{j,r\to s}\sim N(\beta_{r\to s},\sigma_{r\to s}^{2})$$

whose means $\beta_{r\to s}$ were given the same priors as in (6), and have the same properties (2). Regarding the variances $\sigma_{r\to s}^{2}$, we hypothesize that the coefficient of variation (CV) of each mapping, the between-trials SD divided by the mean, is the same on each instrument, where ϕ is the CV:

(8) $$\sigma_{r\to s}^{2}=\beta_{r\to s}^{2}\phi^{2},\;\phi \sim U(0,\;1)$$

The model assumes that each trial samples all *M* = 6 treatment effects from a multivariate normal distribution, but some test instruments are missing at random. As such, the model generates predicted treatment effects $\delta_{jm}=\beta_{1\to m}\delta_{j1}$ on each instrument on each trial, and enables us to report mean treatment effects $\mu_{m}=\beta_{1\to m}\mu_{1}$ and between-trial SDs $\sigma_{m}=Abs(\beta_{1\to m})\sigma_{1}$ on each instrument.

Estimation was carried out by Markov Chain Monte Carlo using WinBUGs [43]. The code and data set are set out in full in Appendix D in Supplemental Materials found at doi:10.1016/j.jval.2013.12.006. Goodness of fit was assessed via the posterior mean residual deviance [44]. The residual deviance for a multivariate normal likelihood is, summing over trials *j*, as follows

(9) $$\bar{D}=\sum_{j}\left({\hat{D}}_{j}-\delta_{j})S_{j}^{-1}({\hat{D}}_{j}-\delta_{j}\right)$$

Model fit is usually considered to be adequate when the posterior mean $\bar{D}$ is approximately equal to the number of data points, 32 in the Table 1 data set. We also calculated the deviance information criteria [44], a measure of goodness of fit penalized by the number of effective parameters. The latter was calculated by calculating goodness of fit at the posterior mean of the outcomes predicted by the model [24]. Further diagnostic checks were carried out: first, we looked at the posterior mean residual deviance, the Mahanobolis distance, for each trial separately, and second, we examined residuals (observed minus predicted treatment effect for every treatment effect, to check that, for each test instrument, the predicted treatment effects were not systematically too high or too low).

Convergence, based on standard statistical criteria [45], occurred within 20,000 in the fixed mapping model, and for most parameters within 30,000 in the random mapping model. One parameter required 80,000 samples to converge. Posterior summaries for both fixed and random mapping models have been based on 100,000 samples from each of five chains, having discarded the first 100,000.

## Results

Posterior summaries of treatment effects on Pain-VAS are shown in Table 3. The mean treatment effect and its precision, and the between-trials variation, are relatively insensitive to whether fixed or random models are chosen for the mapping. The fixed mapping model, however, fitted poorly, with residual deviance $\bar{D}$= 57.6 compared with 34 data points. Globally, the random mapping model fitted adequately with a $\bar{D}$of 35.8. The number of effective parameters in the random mapping model was, unusually, less than in the fixed mapping model. This suggests that the number of additional effective mapping parameters is relatively few and that their presence allows greater “shrinkage” of treatment effects toward their mean value. The deviance information criterion, which is the sum of the residual deviance and the number of parameters, therefore strongly favors the random mapping model.

**Table 3:** Posterior summaries of mapping, treatment effect and variation parameters, and goodness-of-fit statistics under fixed and random mapping models.*

**Parameters**	**Fixed mapping**	**Random mapping**
Treatment effect on Pain-VAS		
Mean, $\mu_{1}$	−2.26 (0.24)	−2.30 (0.25)
Between-study SD, $\sigma_{1}$	0.40 (0.031, 1.04)	0.42 (0.028, 1.10)
Mappings from Pain-VAS to:		
BASFI, $\beta_{1\to 2}$	0.68 (0.038)	0.68 (0.056)
BASDAI, $\beta_{1\to 3}$	0.94 (0.039)	0.92 (0.072)
ASQOL, $\beta_{1\to 4}$	1.21 (0.12)	1.21 (0.28)
SF-36 PCS, $\beta_{1\to 5}$	−2.96 (0.19)	−2.88 (0.30)
SF-36 MCS, $\beta_{1\to 6}$	−0.60 (0.23)	−0.59 (0.24)
CV for mappings, between-study, ϕ	–	0.130 (0.055, 0.25)
Goodness-of-fit statistics		
Residual deviance, $\bar{D}$	57.6	35.8
Effective number of parameters, $p_{D}$	21.3	17.9
Deviance information criterion, DIC	78.8	53.8

ASQOL, Ankylosing Spondylitis Quality of Life scale; BASDAI, Bath Ankylosing Spondylitis Disease Activity Index; BASFI, Bath Ankylosing Spondylitis Functional Index; CV, coefficient of variation; SF-36 MCS, short-form 36 health survey mental component summary; SF-36 PCS, short-form 36 health survey physical component summary; VAS, visual analogue scale.

⁎ For mappings and treatment effect, posterior means (SDs); for SDs, posterior medians (2.5, 97.5 centiles).

The estimated mapping ratios from each instrument to Pain-VAS are also presented in Table 3. It is evident that the mapping model has little effect on their mean value. As might be expected, their posterior precision is somewhat lower in a random mapping model, but even under random mapping they are estimated with a relatively high precision. Furthermore, the degree of variation in mappings from trial to trial is relatively low, with a CV ϕ showing a median value of only 0.13, with an upper (97.5%) credible limit of 0.24. This indicates a between-studies SD that is only, on average, 13% of the mean.

The ability of the method to generate pooled treatment effects on any of the scales is illustrated in Table 4.

**Table 4:** Posterior summaries of treatment effects on each of the instruments, and between-study SDs, under the random mapping model.

	**Mean treatment effect (SD)**${µ}_{1,\;2,\;3,\;4,\;5,\;6}$	**Between-studies mean SD (2.5, 50, 97.5 percentiles)**$\sigma_{1,\;2,\;3,\;4,\;5,\;6}$
Pain-VAS	−2.30 (0.25)	0.42 (0.03, 0.38, 1.10)
BASFI	−1.55 (0.19)	0.28 (0.02, 0.25, 0.75)
BASDAI	−2.11 (0.25)	0.39 (0.03, 0.35, 1.03)
ASQOL	−2.78 (0.70)	0.51 (0.03, 0.44, 1.44)
SF-36 PCS	6.60 (0.89)	1.21 (0.08, 1.07, 3.18)
SF-36 MCS	1.36 (0.59)	0.25 (0.01, 0.20, 0.79)

ASQOL, Ankylosing Spondylitis Quality of Life scale; BASDAI, Bath Ankylosing Spondylitis Disease Activity Index; BASFI, Bath Ankylosing Spondylitis Functional Index; SF-36 MCS, short-form 36 health survey mental component summary; SF-36 PCS, short-form 36 health survey physical component summary; VAS, visual analogue scale.

**Fig. 1:**
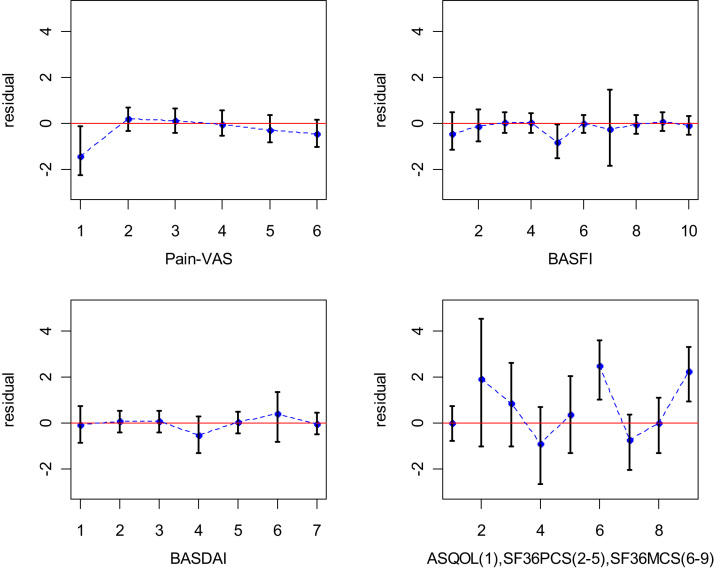
Residuals of estimates for treatment effect measured on six instruments: 1) Pain-VAS; 2) BASFI; 3) BASDAI; 4) ASQOL; 5) SF-36 PCS; and 6) SF-36 MCS under the random mapping model. ASQOL, Ankylosing Spondylitis Quality of Life scale; BASDAI, Bath Ankylosing Spondylitis Disease Activity Index; BASFI, Bath Ankylosing Spondylitis Functional Index; SF-36 MCS, short-form 36 health survey mental component summary; SF-36 PCS, short-form 36 health survey physical component summary; VAS, visual analogue scale.

We developed two model diagnostic procedures. The first (Table 5) looks at the goodness of fit in each trial. The residual mean deviances are compared with their expected values, which is the number of data points. The fit of each trial seems adequate. The second plots the residuals (Fig. 1) and allows us to check that none of the outcomes are associated with a systematic error. We explored the effect of raising or lowering the assumed correlations between change scores on different instruments. Making all the correlations 10% smaller, or 10% greater without changing their sign, had little effect (<1%) on the posterior means of either the treatment effect or the mapping coefficient distributions. Increasing the correlation decreases the total amount of information on the treatment effect but increases the information on mappings, and this is reflected in slight changes in posterior SDs. Changes to the correlation had an effect on mean deviance residual (max 10%), between-trial variation in treatment (10%–20%), and between-trial CV in mappings (7%–10%).

**Table 5:** Residual mean deviance by trial.

**Trial**	**Mean residual deviance**	**Number of data points**
1. Gorman (2002) [25]	4.8	2
2. Brandt (2003) [26]	0.7	2
3. Davis (2003) [27]	3.0	3
4. van der Heijde (2006) [28]	2.2	3
5. Braun (2002) [29]	2.6	4
6. van der Heijde (2005) [30]	4.1	5
7. van der Heijde (2009) [31]	5.9	5
8. Inman (2008) [32]	12.4	8

## Discussion

The method provides a new solution to two problems, hitherto seen as unrelated: multioutcome synthesis and between-outcome mapping. In this section, we begin by focusing attention on specific results obtained with these data, and consider some alternative models that might have been used. We then compare properties of the proposed approach with current methods in the two fields of multioutcome synthesis and mapping. We end with comments on limitations and further research needs.

We assumed a standard random treatment effect model, but other treatment models could have been fitted. A fixed treatment effect model, not shown here, fitted very poorly. An alternative treatment model that could be fitted, if the objective is to compare the efficacy of different biologics, is a network meta-analysis [24]. We would anticipate that mappings, which depend on within-trial information, would be relatively insensitive to treatment effect models, which are driven by between-trial information. Mappings could still be estimated if we assumed that treatment effects in each trial were entirely unrelated, but then pooled treatment effects could not be estimated. Note, however, that mappings between instruments can be accurately estimated only if there is a “network” of connections between them. A trial reporting an “unconnected” instrument could be included, but the between-trial variation in treatment effect would be confounded by the between-trial variation in mapping.

The random mapping model fitted each trial, and we found no signs of systematic deviation from the model. The degree of between-trial variation in mapping ratios will depend entirely on the data. In this data set, although we can definitely reject the null hypothesis that mappings are exactly the same from one trial to the next, the degree of between-trial variation in mappings was relatively small, with a CV of only 13%. This lends credibility to the model. If the CV had been 20%, for example, then, for a mapping with a central value of 2.0, 95% of the studies would have true mappings between 1.22 and 2.78. But, if the CV was as high as, say, 40%, the 95% limits on study-specific mappings would be 0.43 to 3.37, at which point one might begin to question the usefulness of the concept of mapping, and doubt the validity of our structural equation approach (3). In the context of HTA, high precision and lack of variability in mappings is obviously desirable, and also potentially in trial design [35].

A number of methods for the synthesis of multiple outcomes have been proposed, with different objectives and different scope. If different test instruments are considered to measure approximately the same underlying construct, as in tests of depression, or social anxiety, a strategy often adopted in evidence synthesis is to standardize treatment effects by dividing through by the sample SD [11]. This has been strongly criticized [46]. Division by the sample SD, while not contributing to bias, will contribute extra heterogeneity to treatment effects, especially when based on small samples. Second, the population SDs often differ markedly, and trial designers may select a narrow group of patients to increase the probability of detecting a “significant” effect. This will exaggerate standardized effects and introduce further heterogeneity.

Our approach allows reporting the pooled result on any of the original clinical scales, as recommended in the Cochrane Handbook [39], but without the problems created by standardization. Also, unlike standardization, it is possible to incorporate information from a far wider range of tests, not just those apparently measuring the same construct, and without assuming that tests are equally sensitive to treatment, which appears to be an implicit assumption in standardization.

A quite different approach attempts to “borrow strength” across outcomes by fitting multivariate normal models to multiple continuous outcomes [13], [14], [15], [16], [17], [18]. The extent of “borrowing strength” is usually very small unless data are missing on one or more outcomes, in which case these models can improve on the precision of the univariate estimates [14], [15]. The models used here represent a special case of these multivariate normal models in which treatment effects on all outcomes are the same within a constant (fixed mapping), or similar (random mapping). At the within-trial level, both approaches have the same likelihood and estimate the same number of parameters per trial. Whether or not the mapping method borrows strength more effectively between trials awaits a more detailed analysis. It seems likely, however, that this will be the case because there are only two between-trial variance parameters to estimate, rather than one variance parameter per outcome. The critical point, however, is that multivariate normal meta-analysis does not generate mappings at all, let alone mappings that are consistent with treatment effects. Furthermore, if multivariate normal is used and mapping is required, the mapped parameters will not be consistent with the evidence synthesis.

Our proposals also have implications for the common practice of “mapping” an estimated treatment effect on a DSM into an estimated treatment effect on another instrument, usually a generic HRQOL, when the latter has not been measured in a trial [19], [20]. Mappings based on trial-generated treatment effects have not, it appears, been envisaged so far in the literature. Instead mappings have been estimated from cohort studies, usually from cross-sectional data in which patients with the condition in question are assessed on both the DSM and the generic instruments. The resulting estimates are then applied to a DSM treatment effect to provide a treatment effect on the generic HRQOL.

The common use of ordinary least squares regression to estimate mappings in cohort studies has been criticized on grounds that resulting estimates are neither transitive nor invertible [22]. The extensive literature on test equating and aligning (see, e.g., Dorans et al. [47] and Kolen and Brennan [48]) also asserts that invertability is a requirement and that this rules out ordinary least squares. But regardless of the methodology used, one can question whether it is even theoretically *possible* to identify mappings between treatment effects in studies in which causal treatment effects cannot be identified, and in which treatments might not feature at all. As far as we know, the precise assumptions under which this could be achieved have never been elaborated.

Our clear finding of across-study heterogeneity in mappings, although at a relatively low level, runs counter to assumptions implicit in the health economics literature that mappings are constant across studies. As a result, it is likely that the precision attributed to estimated mappings used in HTA has been quite severely exaggerated.

Perhaps a greater weakness in the way mappings are implemented in HTA practice is the reliance placed on mapping from, most commonly, just one DSM to the target generic instrument. In many cases, most of the trial evidence, including treatment effects on the target generic scale itself, is ignored. For ankylosing spondylitis, different mappings, usually various linear equations in Bath Ankylosing Spondylitis Disease Activity Index and Bath Ankylosing Spondylitis Functional Index, derived from either cohorts or trials treated as observational cohorts, have been used in cost-effectiveness models [49]. Inefficient use of trial data, mapping a single DSM into the EQ-5D questionnaire scale when several are available, can be found in economic models of Alzheimer’s disease [50], [51] or psoriatic arthritis [52].

In contrast, use of the trial evidence on *all* trial outcomes, as illustrated here, allows us to pool treatment effect information over all scales, the validity of which is supported here by the low CV, and at the same time provides a joint and unbiased mapping that all investigators can use, which is coherently and transparently derived from the same, noncontroversial, trial-based evidence used to estimate treatment effects themselves. Furthermore, unlike mappings derived from ordinary least squares regression, these approaches will not routinely underestimate effects of treatments on generic HRQOL scales [22].

An important limitation of the method is that it requires that the target HRQOL scale, such as the EQ-5D questionnaire, is one of the outcome measures in a connected network of outcomes. In our example, there is a lack of trial data on the EQ-5D questionnaire, and so no mapping to the EQ-5D questionnaire can be derived. One should remember that not all mapping is based on linear relationships: in some clinical areas, studies estimating mean EQ-5D questionnaire scores in “mild,” “moderate,” and “severe” patients, as defined by a continuous scale, are used to assess the effect of treatment on the EQ-5D questionnaire. There would be advantages to using simultaneous mapping and synthesis of all available DSMs, as advocated here, to estimate the proportion of patients in each severity group following treatment. Alternatively, if the target HRQOL is not part of the network of outcomes, it is open to investigators to use geometric regression [22] or other methods [47], [48] to map between the EQ-5D questionnaire and one of the outcomes in the network.

In applications it will be important to limit sources of heterogeneity that could generate variation in mappings. The assumption of multivariate normality in treatment effects, and hence linearity of treatment effects, is fairly standard for the continuous, or commonly interpreted as continuous, patient- and clinician-reported outcomes for which the method is intended. But there is an implicit assumption of approximately linear relations between the underlying scales at the patient level. If two measures are *not* linearly related, the ratios of mean effects will vary across the measurement spectrum. This will show up as heterogeneity in mapping ratios. This is the likely mechanism of heterogeneity in mappings, and reminds us that all kinds of evidence synthesis give better results in homogeneous sets of patients. Efforts to apply these methods to network meta-analyses [24] involving heterogeneous classes of treatment may also be a source of additional heterogeneity in mappings, if, for example, a subset of the test instruments were more sensitive to particular classes of treatments. The usefulness of these methods will begin to be clear only after they have been applied to a wide range of data sets on different conditions.

Source of financial support: This work has been supported by funding from the Medical Research Council (grant no. G0901488), and by funding from the National Institute for Health and Care Excellence to the Clinical Guidelines Technical Support Unit at the University of Bristol. The authors are grateful to Dr. Jon Packham, Dr. Kirstie Haywood, and other organizers of the Evaluation of Ankylosing Spondylitis Quality of Life study for permission to use the correlation data, and to Dr. Roberta Ara for facilitating this.
