# Supplementary material for: Simultaneous Multioutcome Synthesis and Mapping of Treatment Effects to a Common Scale
Source: Value Health. 2014 Mar;17(2):280–7. doi: 10.1016/j.jval.2013.12.006 (PMC3991420; doi:10.1016/j.jval.2013.12.006)
Supplement: Supplementary file 1 — Supplementary Material [file mmc1.pdf]

SUPPLEMENTARY MATERIAL

APPENDIX A: Calculation of variances of change scores

The data that appears in Table 1 is based on a number of approximations, assumptions, and figures extracted from graphs. Details are given here.

Inman (2008). Mean differences based on median Change from Baseline (CfB) given in Table 2. Standard Errors derived from samples sizes and inter-quartile ranges

Brandt (2003). Follow-up and baseline reported separately. Baseline BASDAI taken from from Figure 4. Standard Errors of CfB assumes correlation between baseline and follow-up scores of 0.5

Gorman (2002). Follow up and baseline reported separately, Standard error of CfB assumes r=0.5. Total Back Pain VAS was not reported; Nocturnal Pain has been used instead.

Van der Heijde (2009). CfB and its standard error from Table 2.

Braun (2003). Mean changes in BASDAI and BASFI reported in text. Standard Deviation (SD) at follow-up based on 95% confidence interval in Figure 3. SD at baseline from Table 1. Standard Error of CfB assumes correlation of 0.5. SF-36 results at baseline and follow-up reported as “percent” in Table 2 were divided by 2 to transform to standard 50 point scale.

Davis (2003) . Mean and standard errors PAIN-VAS at baseline and follow-up were reported on 100-point scales and divided by 10. Standard error of CfB based on correlation of 0.5.

Van der Heijde (2005). Median CfB in Table 2 taken as an estimate of mean CFB. Standard Error of CFB derived from inter-quartile ranges in Table 2

Van der Heijde (2006). Mean CfB and standard Error reported in Table 2. Pain-VAS and BASFI were reported as percent change. These were converted to absolute CFB based on the treatment group mean at baseline (Table 1).

APPENDIX B: Correlations between change scores

This appendix derives the relationship between the correlation ρ_{rs} between change scores on two instruments *r* and *s*, and the correlation η_{rs} between scores observed cross-sectionally. Suppressing the notation *C* or controls and *T* for the treatment groups, as correlations are assumed to be the same in each group, for scores on instruments *r* and *s*, at Baseline (*B*) and Follow-up (*F*), define:

$$D_r = Y_{rF} - Y_{rB}, \quad D_s = Y_{sF} - Y_{sB}$$

Let ρ_{BF} be the correlation between scores at Baseline and Follow-up on both *r* and *s*. Then

$$\begin{aligned} Cov(D_r, D_s) &= Cov\left(Y_{rF} - Y_{rB}, Y_{sF} - Y_{sB}\right) \\ &= Cov\left(Y_{rF}, Y_{sF}\right) + Cov\left(Y_{rB}, Y_{sB}\right) - Cov\left(Y_{rF}, Y_{sB}\right) - Cov\left(Y_{rB}, Y_{sF}\right) \\ &= 2\eta_{rs}\sqrt{V_r V_s} - 2\left(\eta_{rs}\rho_{BF} + k\sqrt{\left(1-\eta_{rs}^2\right)\left(1-\rho_{BF}^2\right)}\right)\sqrt{V_r V_s} \\ &= 2\left(\eta_{rs} - \eta_{rs}\rho_{BF} - k\sqrt{\left(1-\eta_{rs}^2\right)\left(1-\rho_{BF}^2\right)}\right)\sqrt{V_r V_s} \end{aligned} \qquad \text{(B1)}$$

where *k* is a constant lying in the interval [-1,1], and we have assumed that variance does not vary with time.

In addition:

$$\begin{aligned} Var\left(D_r\right) &= Var\left(Y_{rF}\right) + Var\left(Y_{rB}\right) - 2Cov\left(Y_{rF}, Y_{rB}\right) = 2\left(1-\rho_{BF}\right)V_r \\ Var\left(D_s\right) &= Var\left(Y_{sF}\right) + Var\left(Y_{sB}\right) - 2Cov\left(Y_{sF}, Y_{sB}\right) = 2\left(1-\rho_{BF}\right)V_s \end{aligned}$$

Thus, the correlation ρ_{rs} in general case can be written as

$$\begin{aligned} \rho_{rs} &= \frac{2\left(\eta_{rs} - \eta_{rs}\rho_{BF} - k\sqrt{\left(1-\eta_{rs}^2\right)\left(1-\rho_{BF}^2\right)}\right)\sqrt{V_r V_s}}{2\left(1-\rho_{BF}\right)\sqrt{V_r V_s}} \\ &= \frac{\eta_{rs} - \eta_{rs}\rho_{BF} - k\sqrt{\left(1-\eta_{rs}^2\right)\left(1-\rho_{BF}^2\right)}}{1-\rho_{BF}} \end{aligned} \qquad \text{(B2)}$$

To guarantee $|\rho_{rs}| \le 1$, we further require

$$\max\left\{-1, \ -\sqrt{\frac{\left(1-\eta_{rs}\right)\left(1-\rho_{BF}\right)}{\left(1+\eta_{rs}\right)\left(1+\rho_{BF}\right)}}\right\} \le k \le \min\left\{1, \ \sqrt{\frac{\left(1+\eta_{rs}\right)\left(1-\rho_{BF}\right)}{\left(1-\eta_{rs}\right)\left(1+\rho_{BF}\right)}}\right\}$$

We now assume that the causal path is $Y_{rB} \to Y_{sB} \to Y_{sF}$, i.e. $Corr\left(Y_{rB}, Y_{sF} \mid Y_{sB}\right) = 0$, then k=0, and $Corr\left(Y_{rB}, Y_{sF}\right) = \eta_{rs}\rho_{BF}$, implying $Cov\left(D_r, D_s\right) = 2\eta_{rs}\left(1-\rho_{BF}\right)\sqrt{V_r V_s}$ from (B1), and from (B2) $\rho_{rs} = \eta_{rs}$.

We examine sensitivity to the k=0 assumption by raising or lowering $Abs(\rho_{rs})$ by 10%.

APPENDIX C. Covariances in multi-arm trials

Here we derive expressions for the variances and covariances between treatment effects on different instruments in multi-arm trials.

A trial with *M* outcomes and *K* arms, one of which is placebo, will estimate *M*(*K-1*) effects relative to placebo, with a variance-covariance matrix with $[M(K\text{-}1)]^2$ terms. For example, a 3-arm trial comparing treatments *k* and *h* to placebo *C,* and reporting outcomes *r* and *s*, we need the covariance of treatment effects on each instrument in different arms, and the covariance of treatment effects on different instruments in different arms. Where $\bar{Y}_{rk}, \bar{Y}_{rh}, \bar{Y}_{rC}$ are the mean change scores in arms *k, h, C* on instrument *r,* and $\bar{Y}_{sk}, \bar{Y}_{sh}, \bar{Y}_{sC}$ are the mean change scores on instrument *s,* using an approach analogous to Franchini (2012) and Wei (2012), we can find:

$$Cov(\hat{D}_{rk}, \hat{D}_{rh}) = Cov(\bar{Y}_{rk} - \bar{Y}_{rC}, \bar{Y}_{rh} - \bar{Y}_{rC}) = \frac{V_{rC}}{n_C}; \quad \text{and}$$

$$Cov(\hat{D}_{sk}, \hat{D}_{sh}) = Cov(\bar{Y}_{sk} - \bar{Y}_{sC}, \bar{Y}_{sh} - \bar{Y}_{sC}) = \frac{V_{sC}}{n_C}$$

$$\begin{aligned} Cov(\hat{D}_{rk}, \hat{D}_{sh}) &= Cov(\bar{Y}_{rk} - \bar{Y}_{rC}, \bar{Y}_{sh} - \bar{Y}_{sC}) \\ &= Cov(\bar{Y}_{rk}, \bar{Y}_{sh}) - Cov(\bar{Y}_{rk}, \bar{Y}_{sC}) - Cov(\bar{Y}_{rC}, \bar{Y}_{sk}) + Cov(\bar{Y}_{rC}, \bar{Y}_{sC}) \\ &= \rho_{rs} \frac{\sqrt{V_{rC} V_{sC}}}{n_C}, \end{aligned}$$

References

Franchini AJ, Dias S, Ades AE, Jansen JP, Welton NJ. Accounting for correlation in network meta-analysis in multi-arm trials. *Research Synthesis Methods* 2012; 3: 142-160.

Wei Y, Higgins J. Estimating within-study covariances in multivariate meta-analysis with multiple outcomes. *Statistics in Medicine* 2012; 32:1191-1205

APPENDIX D

Here we give annotated WinBUGS code, dataset and initial values for the Random Mapping model. WinBUGS does not allow multi-variate normal distributions with varying numbers of dimensions. For this reason the separate likelihoods are required for each block of trials depending on the number of test instruments used, and the number of arms. The one 3-arm trial, which has four outcomes reported, has an 8x8 variance-covariance matrix.

```
# Random Mapping model
model {
# Random treatment effects model for seven 2-arm trials
for(i in 1:7) {  delta[i]~dnorm(mu, tau)  }     #delta[i] is trt effect in trial i on outcome 1

# Random treatment effects model for one three arm trial
for(j in 1:2) {a[j] ~ dnorm(0,tau) }
ddelta[1]<- mu + 0.5*(sqrt(3)*a[1]+a[2])
ddelta[2]<- mu + 0.5*(sqrt(3)*a[1]-a[2])

beta.m[1]<-1
for(k in 2:6) {    beta.m[k]~dnorm(1,.01)  }              #non-info normal priors for beta.m[k]
for(k in 2:6) {      tau.m[k]<-1/pow(sig.m[k],2)
                    sig.m[k]<-abs(beta.m[k])*sig.m1 }    #common sig.m1     }
for (k in 1:6)  {sbeta.m[k] <- sign[k] * abs(beta.m[k]) }

 for(i in 1:8) {                        # 8 trials
      beta[i,1]<-1
      for(k in 2:6) { beta[i,k] ~ dnorm(beta.m[k], tau.m[k])  }    #  tau.m varies with k
      for(k in 1:6) { sbeta[i,k] <- sign[k] * abs(beta[i,k])  }        # signed mappings
       }

     sig.m1~dunif(0,1)               # prior for between-trials co-efficient of variation
     mu~dnorm(0,.001)               #  prior for mean treatment effect
     tau<-1/pow(sig,2)
     sig~dnorm(0,10) I(0,)          # prior for between trials sd of treatment effects

# generate mean treatment effects on every instrument
# ... and between-trial sds on every instrument
for (k in 2:6) {mm[k] <- mu * sbeta.m[k]
                  sd[k] <- sig * abs(beta.m[k])   }


# Likelihoods, deviance, and residuals
 #  each block of code sets up a multi-variate normal distribution for the data,
 #        defines the mean and precision for the distribution,
 #        defines the variance and covariance terms, so that precision can be calculated,
 #        monitors the residuals,
 #        and calculates the contribution to residual deviance
# TWO ARM TRIALS
#=1-2 (2-dim)
for(i in 1:2){
     d[i,1:2]~dmnorm(mu2[i,1:2], prec2[i,1:2,1:2])
     prec2[i,1:2,1:2]<-inverse(w2[i,1:2,1:2])
     for(j in 1:2){
```

```
                mu2[i,j]<- delta[i ]* sbeta[i,t[i,j]]
                w2[i,j,j]<-(vC[i,j]/nC[i]) + (vT[i,j]/nT[i])}
       for(k in 1:1){
            for(h in (k+1):2) {w2[i,k, h]<- (sqrt(vC[i,k]*vC[i,h])/nC[i] +
                                        sqrt(vT[i,h]*vT[i,k])/nT[i])  *  C1[t[i,k],t[i,h]]
                              w2[i,h, k]<-w2[i,k, h] }}
       for(k in 1:2){ res[i, k]<-d[i,k]-mu2[i,k] }
       for(k in 1:2){  m[i,k]<-inprod(prec2[i,k,1:2], res[i,1:2]) }
       dev[i]<-inprod(m[i,1:2],res[i,1:2])
     }
```

#=3-4 (3-dim)

```
for(i in 3:4){
      d[i,1:3]~dmnorm(mu3[i,1:3], prec3[i,1:3,1:3])
      prec3[i,1:3,1:3]<-inverse(w3[i,1:3,1:3])
      for(j in 1:3){
                mu3[i,j]<- delta[i ]* sbeta[i,t[i,j]]
                w3[i,j,j]<-(vC[i,j]/nC[i]) + (vT[i,j]/nT[i])}
       for(k in 1:2){
            for(h in (k+1):3){w3[i,k, h]<- (sqrt(vC[i,k]*vC[i,h])/nC[i] +
                                          sqrt(vT[i,h]*vT[i,k])/nT[i])  * C1[t[i,k],t[i,h]]
                             w3[i,h, k]<-w3[i,k, h] }}
       for(k in 1:3){ res[i, k]<-d[i,k]-mu3[i,k] }
       for(k in 1:3){  m[i,k]<-inprod(prec3[i,k,1:3], res[i,1:3]) }
       dev[i]<-inprod(m[i,1:3],res[i,1:3])
     }
```

#=5-5 (4-dim)

```
for(i in 5:5){
      d[i,1:4]~dmnorm(mu4[i,1:4], prec4[i,1:4,1:4])
      prec4[i,1:4,1:4]<-inverse(w4[i,1:4,1:4])
      for(j in 1:4){
                mu4[i,j]<- delta[i ]* sbeta[i,t[i,j]]
                w4[i,j,j]<-(vC[i,j]/nC[i]) + (vT[i,j]/nT[i])}
       for(k in 1:3){
            for(h in (k+1):4){w4[i,k,h] <- (sqrt(vC[i,k]*vC[i,h])/nC[i] +
                                          sqrt(vT[i,h]*vT[i,k])/nT[i])  * C1[t[i,k],t[i,h]]
                             w4[i,h,k] <- w4[i,k,h] }}
       for(k in 1:4) { res[i,k] <- d[i,k]-mu4[i,k] }
       for(k in 1:4) { m[i,k] <- inprod(prec4[i,k,1:4], res[i,1:4]) }
       dev[i]<-inprod(m[i,1:4],res[i,1:4])
     }
```

#=6-7 (5-dim)

```
for (i in 6:7) {
      d[i,1:5] ~ dmnorm(mu5[i,1:5], prec5[i,1:5,1:5])
      prec5[i, 1:5,1:5]<-inverse(w5[i,1:5,1:5])
      for(j in 1:5){
                mu5[i,j] <- delta[i ]* sbeta[i,t[i,j]]
                w5[i, j,j] <- (vC[i,j]/nC[i]) + (vT[i,j]/nT[i])}
       for(k in 1:4){
            for(h in (k+1):5) {w5[i,k,h] <- (sqrt(vC[i,k]*vC[i,h])/nC[i] +
                                          sqrt(vT[i,h]*vT[i,k])/nT[i] ) * C1[t[i,k],t[i,h]]
                             w5[i,h,k] <- w5[i,k,h] }}
       for(k in 1:5)  { res[i,k] <- d[i,k]-mu5[i,k] }
       for(k in 1:5)  { m[i,k] <- inprod(prec5[i,k,1:5], res[i,1:5]) }
       dev[i]<-inprod(m[i,1:5],res[i,1:5])
       }
```

# THREE-ARM TRIALS

#=8-8 (4-dim, 3-arm) on instruments 1,2, 5, 6

```
d2[1:8] ~ dmnorm(mu42[1:8], prec42[1:8,1:8])
prec42[1:8,1:8] <- inverse(w42[1:8,1:8])

#  diagonal terms 11;22;33;......88
for (j in 1:4) { for (k in 1:2) {
    mu42[j + 4*(k-1)] <- ddelta[k] * sbeta[8,t2[j]]            #  means
    w42[j + 4*(k-1),j + 4*(k-1)] <- (vC2[j]/nC2) + (vT2[j + 4*(k-1)]/nT2[k])  }}

# cov between outcomes, same arm
# [1] for 12;13;14;23;24;34
for (k in 1:3) { for (h in (k+1):4) {
    w42[k,h] <- (sqrt(vC2[k]*vC2[h])/nC2 +sqrt(vT2[h]*vT2[k])/nT2[1])  *
                 C1[t2[k],t2[h]]  }}
# [2] for 56;57;58;67;68;78
for (k in 5:7) { for(h in (k+1):8)  {
    w42[k,h] <- (sqrt(vC2[k-4]*vC2[h-4])/nC2 +sqrt(vT2[h]*vT2[k])/nT2[2]) *
                 C1[t2[k],t2[h]]   }}

#  cov between arms, same outcomes 15;26;37;48
for (k in 1:4) { w42[k,k+4] <- vC2[k] / nC2 }

# cov btwn outcomes diff arms 16;17;18;27;28;38
for (k in 1:3) {  for (h in (5+k):8)  {
    w42[k,h] <- (sqrt(vC2[k]*vC2[h-4]) / nC2 ) *  C1[t2[k],t2[h]] }}

# cov between outcomes diff arms  25;35;36;45;46;47
for (k in 2:4) { for (h in 5:(k+3))  {
    w42[k,h] <- (sqrt(vC2[k]*vC2[h-4]) / nC2) * C1[t2[k],t2[h]] }}

# fill out rest of matrix
for (k in 1:7) { for (h in (k+1):8)  {  w42[h,k] <- w42[k,h]   }}


for(k in 1:8)  { res[8,k]<-d2[k] - mu42[k] }
for(k in 1:8) {  m[8,k]<-inprod(prec42[k,1:8], res[8,1:8]) }
dev[8]<-inprod(m[8,1:8],res[8,1:8])

dev.total<-sum(dev[])                  # total residual deviance
}
```

Initial Values, 5 sets

```
list(mu=-2,sig=.3, beta.m=c(NA,1,1,1,   -1,-1), delta=c(-2,-2,-2,-2,  -2,-2,-2), a=c(-2,-2), sig.m1=0.1)
list(mu=-1,sig=1, beta.m=c(NA,2,2,2,   -2,-2), delta=c(-1,-1,-1,-1,  -1,-1,-1), a=c(-1,-1), sig.m1=0.03  )
list(mu=-1,sig=.5, beta.m=c(NA,1,2,3,  -1,-1), delta=c(-1,-3,-1,-1,   -1,-1,-2), a=c(-1,-2), sig.m1=0.2  )
list(mu=-1.5,sig=.1, beta.m=c(NA,2,1,3,   -2,-1), delta=c(-1,-3,-1,-1,    -1,-1,-2), a=c(-1,-2), sig.m1=0.05  )
list(mu=-3,sig=.05, beta.m=c(NA,3,3,3,  -3,-3), delta=c(-3,-2,-3,-3,  -2,-3,-3), a=c(-3,-1), sig.m1=0.2  )
```

Data

Treatment effects d & sd come from RCTs, correlations come from Cohort study

```
direction of effect:    1  2   3  4  5  6
                        -  -   -  -  +  +
#  1=PainVAS, 2=BASFI, 3=BASDAI,  4=ASQOL,  5= SF36PCS, 6=SF36MCS,
```

rearranged according to number of tests in study

```
d[,1]  d[,2]   d[,3]   d[,4]  d[,5]  t[,1]  t[,2]  t[,3]  t[,4]  t[,5]
# 2-ARM TRIALS
# TWO OUTCOMES
-4.15  -2.2    NA     NA    NA    1      2     NA   NA  NA       # Gorman
-1.7    -2.2    NA    NA    NA     2      3    NA   NA  NA      # Brandt
# THREE OUTCOMES
-1.83  -1.41   -1.91   NA   NA     1      2     3    NA   NA    #  Davis
-1.956  -1.414 -1.8   NA   NA      1      2     3    NA   NA    # Van d Heijde 2006
# FOUR OUTCOMES
-2.4    -2.6     8.4  3.85   NA     2      3     5     6      NA    # Braun
#FIVE OUTCOMES
-1.5    -1.8    - 2.4   5.2    1.6     2      3     4     5     6      # Van d Heijde 2009
-2.6   -1.7     -2.5    9.4    .7      1      2     3    5     6       # Van d Heijde 2005
END
```

```
nC[]  nT[]  vC[,1]   vC[,2]   vC[,3]  vC[,4]  vC[,5]    vT[,1]  vT[,2]     vT[,3]  vT[,4]   vT[,5]
#  2-ARM TRIALS
# TWO OUTCOMES
20     20   7.0959   7.75       NA     NA      NA      5.8093   4.41    NA     NA      NA
16     14   5.53     4.39       NA      NA      NA      3.00      2.77    NA     NA      NA
 # THREE OUTCOMES
139  138  5.936   5.546   4.4764     NA      NA     6.436    5.715  4.8503  NA     NA
107  208  7.6850 5.3688    4.28      NA      NA     8.1874   4.4095   8.32    NA     NA
# FOUR OUTCOMES
35    34    20.07  6.84     148.23  84.258    NA    13.24   13.03   138.02  79.023  NA
#FIVE OUTCOMES
107  208  3.862  5.6603  17.12   75.499 96.568   4.0768 6.0112  16.307  72.405 93.371
78   201   3.7147 2.1980 2.4133 34.295 56.056   12.661 4.9456  8.7922  95.747 75.223
END
```

```
# 3-ARM TRIALS
# FOUR OUTCOMES
list(d2=c(-2.7,   -1.5,     4.9,    1.4,    -2.8,   -1.6,     6.0,     3.6 ),     # Inman 2008
     t2=c( 1,        2,        5,       6,        1,        2,      5,       6    ))
```

```
# 3-ARM TRIALS
# FOUR OUTCOMES
list(nC2=78, nT2=c(138,140),
vC2=c( 6.3523,  2.6596,  46.511, 59.643),
vT2=c( 12.139,  4.9456,  104.65, 54.951,  13.738,  5.2808, 76.514,  128.63),
sign=c(1,1,1,1,-1,-1))
```

corr matrix

```
     C1[,1]        C1[,2]       C1[,3]       C1[,4]       C1[,5]       C1[,6]
 1.0000000  0.7031126  0.8522129   0.7376433 -0.6684237  -0.4929236
 0.7031126  1.0000000  0.8106057   0.8293715 -0.8416082  -0.4627976
 0.8522129  0.8106057  1.0000000   0.8559469 -0.7510928  -0.5833274
 0.7376433  0.8293715  0.8559469   1.0000000 -0.7846521  -0.6543728
-0.6684237 -0.8416082 -0.7510928   -0.7846521  1.0000000  0.3389881
-0.4929236 -0.4627976 -0.5833274   -0.6543728  0.3389881  1.0000000
END
```
